# Association between matrix metalloproteinase-1 (MMP-1) protein level and the risk of rheumatoid arthritis and osteoarthritis: a meta-analysis

**DOI:** 10.1590/1414-431X202010366

**Published:** 2020-12-09

**Authors:** Maopeng Wang, You Zhou, Wei Huang, Yong Zeng, Xinzhi Li

**Affiliations:** Department of Orthopedics, Affiliated Renhe Hospital of China Three Gorges University, Yichang, Hubei, China

**Keywords:** Matrix metalloproteinase-1, Rheumatoid arthritis, Knee osteoarthritis, Susceptibility, Cartilage, Synovial fluid

## Abstract

Recent publications have investigated the potential role of the protein level of matrix metalloproteinase-1 (MMP-1) in the susceptibility to rheumatoid arthritis (RA) and osteoarthritis (OA). However, no unanimous conclusion was obtained. Therefore, we carried out a meta-analysis to explore the association between MMP-1 expression and these two clinical disorders. After database searching and screening, we enrolled a total of eighteen articles for the pooled analysis. We observed a significant association between RA cases and controls in the whole population [SMD (standard mean difference)=1.01, P=0.017]. There were similar positive results in the subgroup analysis of “population-based control” (SMD=1.50, P=0.032) and “synovial fluid” (SMD=1.32, P=0.049). In addition, we observed an increased risk in OA cases, compared with controls, in the overall analysis (SMD=0.47, P=0.004) and subsequent subgroup analysis of “knee OA” (SMD=0.86, P<0.001), “Asian/China” (SMD=0.76, P=0.003), “cartilage-Asian/China” (SMD=1.21, P<0.001), and “synovial fluid-Asian/China” (SMD=0.73, P=0.004). In summary, a high protein level of MMP-1 in synovial fluid may be associated with the susceptibility to RA, and the high MMP-1 level in the cartilage tissue or synovial fluid may be related to the pathogenesis of knee OA in the Chinese population. This should be confirmed by larger sample sizes.

## Introduction

Rheumatoid arthritis (RA), a kind of systemic and chronic autoimmune inflammatory disorder, can affect multiple joints and lead to bone erosion and cartilage degradation (1). A series of factors, such as genetic variants, environment, pro-inflammatory cytokines or chemokines, and relevant signal transduction pathways, contribute to the pathogenesis of RA (2,3). Matrix metalloproteinases (MMPs), a family of zinc-dependent endopeptidases, can facilitate extracellular matrix degradation and arterial tissue remodeling during a series of physiological or pathological processes (4,5). In this study, we aimed to analyze the effect of the matrix metalloproteinase-1 (MMP-1) level in the serum or synovial fluid on the risk of RA.

Osteoarthritis (OA), a degenerative multifactorial joint disorder, mainly affects knee joints and can result in deeply damaged and irreversible morphological changes of articular cartilage, body pain, and loss of function (6–9). Factors of age, obesity, genetic variant, cytokines, and extracellular vesicles are implicated in the pathophysiology of OA (3,6,10). The alteration of MMPs in synovial fluid and serum may be linked to the pathogenesis of OA in an animal model (11).

To date, there is still no meta-analysis regarding the correlation between MMP-1 expression and the risk of RA. Furthermore, there is only one meta-analysis published in 2015 that investigated the potential role of MMP-1 expression in the OA susceptibility (12). In the present study, we pooled the available evidence (updated to July 2020) for a comprehensive assessment regarding the potential effect of the MMP-1 level on the risk of RA and OA.

## Material and Methods

### Study identification

We designed our analysis flowchart according to the guidelines of PRISMA (Preferred Reporting Items for Systematic Reviews and Meta-Analyses) (13). Referring to the retrieval strategy of several published meta-analyses (14–16), two investigators performed the publication search for case-control studies from three databases, including Pubmed, Embase (Excerpta Medica Database), and WANFANG, until July 15, 2020. As an example, the search terms of Pubmed were: ((Arthritis, Rheumatoid) OR rheumatoid arthritis)) OR (Osteoarthritis) OR Osteoarthritides) OR Osteoarthrosis) OR Osteoarthroses) OR Arthritis, Degenerative) OR Arthritides, Degenerative) OR Degenerative Arthritides) OR Degenerative Arthritis) OR Osteoarthrosis Deformans)) AND (Matrix Metalloproteinase 1) OR Metalloproteinase 1, Matrix) OR Interstitial Collagenase) OR MMP1 Metalloproteinase) OR Metalloproteinase, MMP1) OR MMP-1 Metalloproteinase) OR MMP 1 Metalloproteinase) OR Metalloproteinase, MMP-1) OR Matrix Metalloproteinase-1) OR Pro-Matrix Metalloproteinase-1) OR Metalloproteinase-1, Pro-Matrix) OR Pro Matrix Metalloproteinase 1) OR Promatrixmetalloproteinase-1) OR Promatrixmetalloproteinase 1) OR proMMP-1) OR MMP1) OR MMP-1). We then designed a series of screening terms, including other diseases or genes. Studies based on meetings, meta-analysis, or review, cell data, animal data, trials, or case reports were excluded.

### Data extraction

After screening, we tried to extract the basic information [e.g., first author, year, region, ethnicity, sample source, sample size, mean value, standard deviation (SD) value, assay, etc.] from the selected full-text articles. Articles with unavailable data were removed. In addition, after the evaluation of the Newcastle-Ottawa Scale (NOS) system, we excluded the studies with poor quality (NOS score ≤5).

### Statistical analysis

We used the STATA software (version 12.0, USA) to perform the statistical analysis. In the Cohen statistics of our association test, the P_A_ (P value of association), SMD (standard mean difference), and 95%CI (confidence intervals) were calculated, under the random-effect model (I^2^>50% or P value of Cochran's Q statistic <0.05). Apart from the overall meta-analysis, we also performed the subgroup analyses by factors of ethnicity (Caucasian/Asian), control source [PB (population-based control)/HB (hospital-based control)], and sample source (serum, synovial fluid, cartilage).

Additionally, a sensitivity analysis was performed to assess the stability of pooling results. Egger's test (Egger's publication bias plot) was also carried out to evaluate whether a remarkable publication bias existed.

## Results

### Included case-control studies

**Figure 1:**
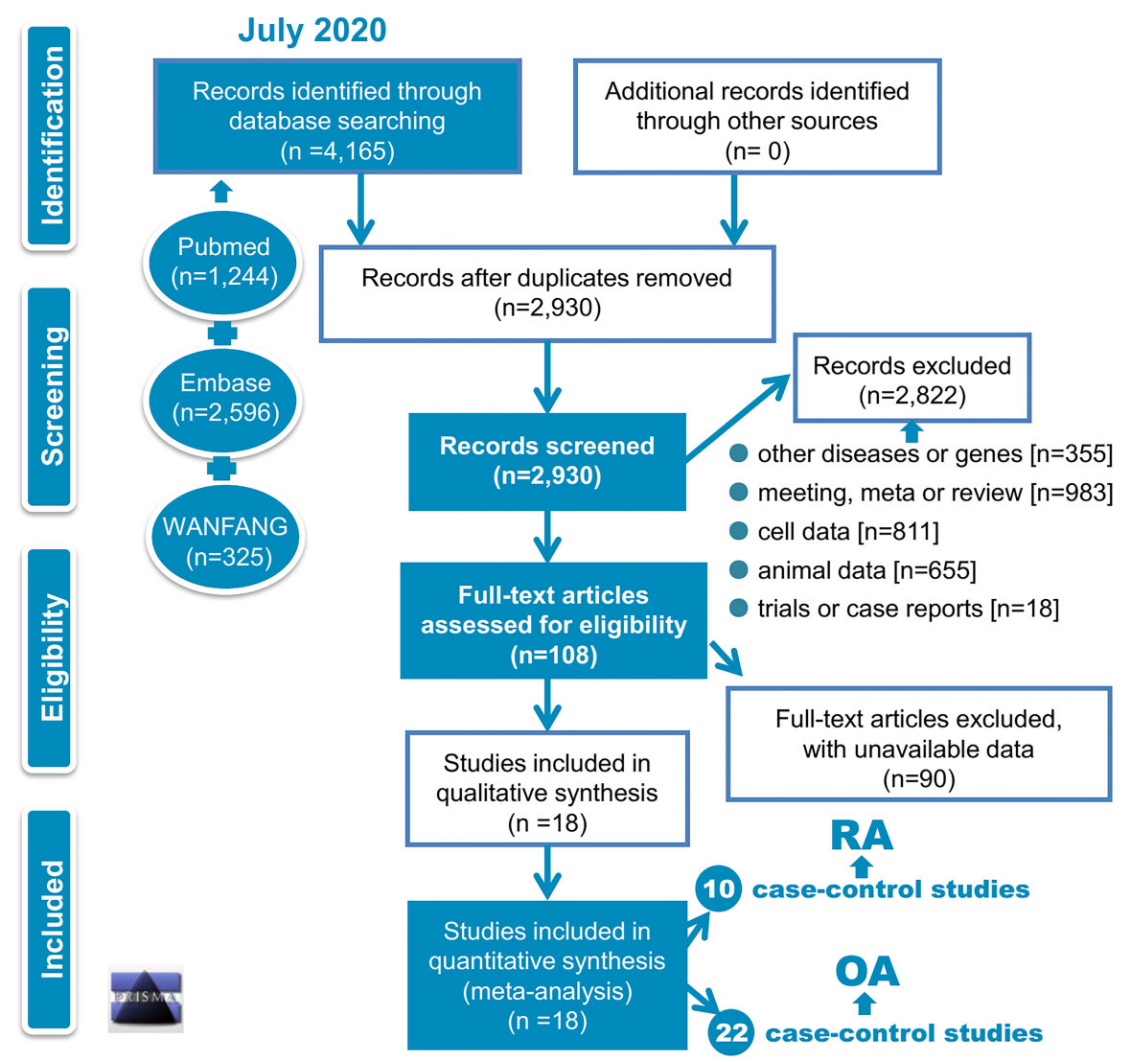
Flowchart of study identification. RA: rheumatoid arthritis; OA: osteoarthritis.

Based on the PRISMA-based flowchart (Figure 1), we included the eligible case-control studies. Briefly, the database search obtained 4,165 records [Pubmed (n=1,244), Embase (n=2,596), WANFANG (n=325)]. Then, we removed 1,235 duplicate records and screened the remaining 2,930 records using our exclusion criteria. A total of 2,822 records were removed with detailed reasons in Figure 1. Next, 108 full-text articles were assessed for eligibility. After the exclusion of 90 articles with unavailable data, eighteen articles (17–34) with high quality were finally identified. Of them, ten case-control studies were included in the meta-analysis of RA, while twenty-two case-control studies were included in that of OA. We present the basic information in Supplementary Table S1.

### Pooling analysis of MMP-1 level and RA risk

**Figure 2:**
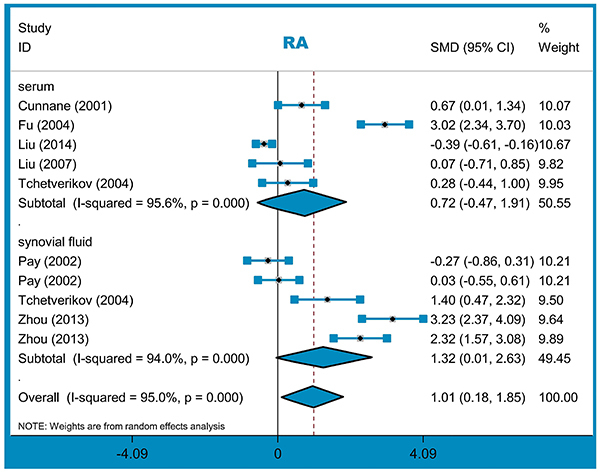
Subgroup analysis by sample source for rheumatoid arthritis (RA) risk. See Supplementary Table S1 for reference numbers.

A total of ten studies (417 cases/332 controls) were enrolled for the meta-analysis of RA. There was an increased risk in the RA cases, compared with controls, in the overall pooling analysis and the subgroup analysis of “PB” and “synovial fluid” (Table 1, all SMD>0, P_A_<0.05). We did not detect a significant association for the other subgroups (Table 1, all P_A_>0.05). Figure 2 presents the forest plot of subgroup analysis by sample source. Therefore, a high MMP-1 level in synovial fluid may be associated with the susceptibility to RA.

**Table 1:** Pooling analysis of matrix metalloproteinase-1 level and rheumatoid arthritis risk.

Group	Studies (n) [cases (n)/controls (n)]	SMD (95%CI)	P_A_	I^2^ (%)	P_H_
Overall	10 (417/332)	1.01 (0.18~1.85)	0.017	95.0%	<0.001
PB	6 (340/256)	1.50 (0.13~2.88)	0.032	97.0%	<0.001
HB	4 (77/66)	0.23 (-0.38~0.84)	0.459	67.3%	0.027
Asian/China	5 (250/248)	1.64 (-0.08~3.35)	0.062	97.6%	<0.001
Caucasian	5 (167/74)	0.35 (-0.15~0.86)	0.170	63.9%	0.026
Serum	5 (308/219)	0.72 (-0.47~1.91)	0.235	95.6%	<0.001
Serum-PB	4 (298/202)	0.88 (-0.59~2.35)	0.240	96.7%	<0.001
Serum-Asian/China	3 (208/194)	0.89 (-1.20~2.98)	0.404	97.7%	<0.001
Synovial fluid	5 (109/103)	1.32 (0.01~2.63)	0.049	94.0%	0.026

PB: population-based control; HB: hospital-based control; SMD: standard mean difference; CI: confidence interval; P_A_: P value from the association test; P_H_: P value from the heterogeneity test.

### Pooling analysis of MMP-1 level and OA risk

**Figure 3:**
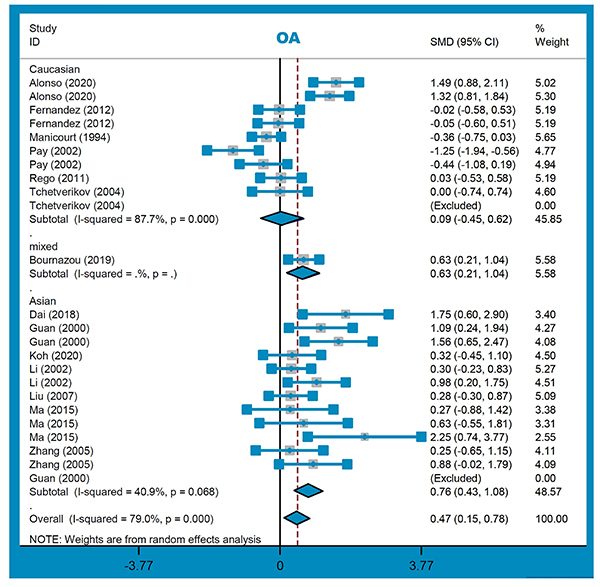
Subgroup analysis by ethnicity for osteoarthritis (OA) risk. See Supplementary Table S1 for reference numbers.

**Figure 4:**
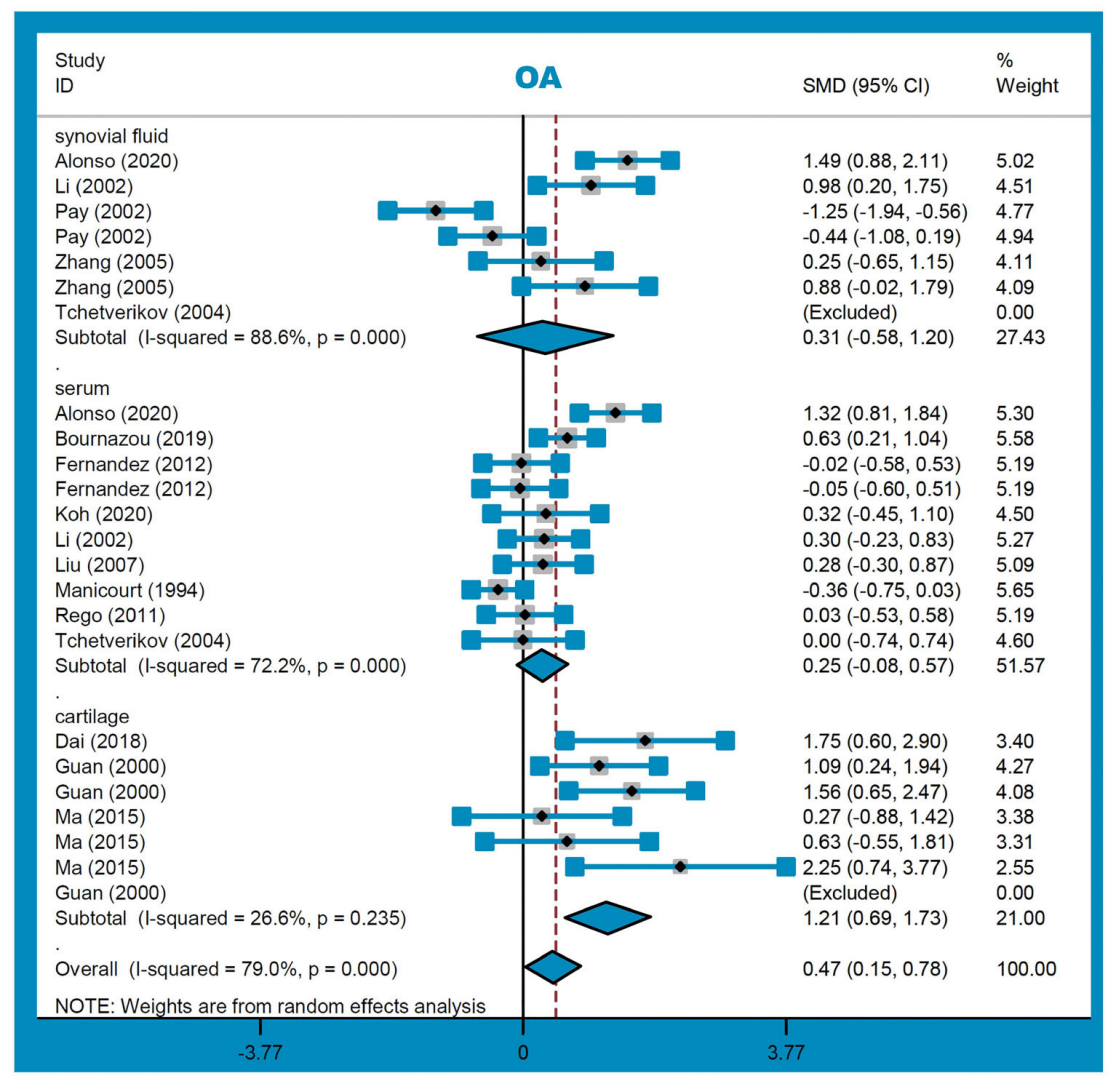
Subgroup analysis by sample source for osteoarthritis (OA) risk. See Supplementary Table S1 for reference numbers.

Twenty-two studies (863 cases/433 controls) were enrolled for the meta-analysis of OA. As presented in Table 2, there was an increased risk in OA cases, compared with controls, in the overall meta-analysis and the following subgroup analysis of knee osteoarthritis “KOA”, “PB”, “HB”, “Asian/China”, “cartilage-HB/KOA/Asian/China”, and “synovial fluid-HB/KOA/Asian/China” (Table 2, all SMD>0, P_A_<0.05). Figures 3 and 4 present the forest plots of subgroup analyses by ethnicity and sample source. These indicated the potential correlation between the high MMP-1 level in the cartilage tissue or synovial fluid and the susceptibility to KOA cases in China.

**Table 2:** Pooling analysis of matrix metalloproteinase-1 level and osteoarthritis risk.

Group	Studies (n) [cases (n)/controls (n)]	SMD (95%CI)	P_A_	I^2^ (%)	P_H_
Overall	22 (863/433)	0.47 (0.15~0.78)	0.004	79.0%	<0.001
KOA	15 (700/189)	0.86 (0.58~1.15)	<0.001	52.0%	0.010
PB	8 (586/256)	0.16 (-0.14~0.46)	0.014	60.4%	0.302
HB	14 (274/177)	0.69 (-0.19~1.19)	<0.001	81.6%	0.007
Asian/China	12 (254/112)	0.76 (0.43~1.08)	0.003	40.9%	0.068
Caucasian	9 (237/297)	-0.09 (-0.45~0.62)	0.752	87.7%	<0.001
Cartilage-HB/KOA/Asian/China	6 (65/42)	1.21 (0.69~1.73)	<0.001	26.6%	0.235
Serum	10 (626/311)	0.25 (-0.08~0.57)	0.135	72.2%	<0.001
Serum-PB	7 (543/248)	0.08 (-0.21~0.37)	0.583	75.8%	0.016
Serum-Caucasian	6 (162/237)	0.15 (-0.36~0.66)	0.562	81.7%	<0.001
Synovial fluid	6 (172/93)	0.31 (-0.58~1.20)	0.492	88.6%	<0.001
Synovial fluid-HB	5 (126/85)	0.18 (-0.86~1.22)	0.732	90.1%	<0.001
Synovial fluid-HB/KOA/Asian/China	3 (97/20)	0.73 (0.24~1.23)	0.004	0.0%	0.456
Synovial fluid-Caucasian	3 (75/73)	-0.06 (-1.67~1.55)	0.940	94.7%	<0.001

KOA: knee osteoarthritis; PB: population-based control; HB: hospital-based control; SMD: standard mean difference; CI: confidence interval; P_A_: P value from the association test; P_H_: P value from the heterogeneity test.

### Heterogeneity analysis

There was a high heterogeneity in the meta-analyses of RA and OA (Tables 1 and 2, all I^2^>50.0%, P_H_<0.001), which led to the use of a random effect model. During the pooled analyses of RA, we observed a reduced heterogeneity in the subgroups of “Caucasian” and “HB” (Table 1), compared with that in the overall meta-analysis. For OA, a similar decreased heterogeneity level was detected in subgroup analyses of “KOA”, “PB”, “Asian/China”, “cartilage-HB/KOA/Asian/China”, and “synovial fluid-Asian/China” (Table 2). Thus, complicating factors, including sample source, control source, ethnic background, may contribute to the high heterogeneity among the included case-control studies.

### Sensitivity and publication bias

**Figure 5:**
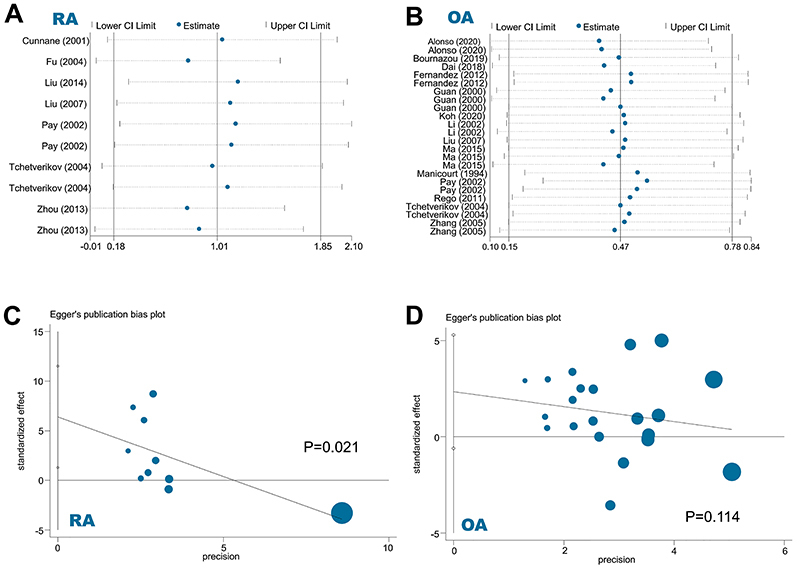
Aand **B**, sensitivity analysis; **C** and **D**, Egger's publication bias plot data. RA: rheumatoid arthritis; OA: osteoarthritis. See Supplementary Table S1 for reference numbers.

The results of sensitivity analysis (Figure 5A for RA; Figure 5B for OA) suggested the data reliability. In addition, we present the Egger's publication bias plots for RA (Figure 5C) and OA (Figure 5D). The absence of significant publication bias was considered for the meta-analysis of OA risk (Figure 5D, P=0.114), but not RA risk (Figure 5C, P=0.021).

## Discussion

There are several studies regarding the role of MMP-1 expression level from the synovial fluid, serum, or cartilage tissue in the risk of OA. For instance, the MMP-1 level of the synovial fluid, but not serum, in OA cases in China is higher than that in controls (25). However, there is a low synovial MMP-1 level in the OA cases in Turkey compared with the controls of other non-RA diseases, including Behcet's disease and familial Mediterranean fever (30).

In 2015, Zeng et al. (12) enrolled seven case-control studies for the first meta-analysis of MMP-1 expression level and the risk of OA, and reported the potential correlation between high MMP-1 level in the synovial joint fluid and OA pathogenesis in the Asian population. In the present study, we included a total of 22 case-control studies for an updated meta-analysis and observed a similar conclusion regarding the effect of MMP-1 level of synovial fluid on the susceptibility to knee OA cases in China. Our findings also indicated that the high protein level of MMP-1 in the cartilage tissue might be linked to the high knee osteoarthritis risk in the Chinese population. In 2018, Geng et al. (35) reported the potential link between MMP-1 rs1799750 and high susceptibility to knee OA cases in China. Recently, two published meta-analyses suggested that MMP-1 rs1799750 may be associated with risk of younger OA cases (<60 years old) (36,37). It is meaningful to investigate the potential effect of MMP-1 protein level on knee OA risk in the Chinese Han population, from the perspective of gene polymorphism. In addition, the factor of age should be fully considered.

Likewise, there are inconsistent conclusions regarding the effect of MMP-1 level of synovial fluid or serum on the risk of RA. For instance, the high MMP-1 level in the synovial fluid is linked to the risk of RA in China (34). However, no increased or decreased synovial MMP-1 level was detected in the RA cases in Turkey compared with the controls of other non-RA diseases (30 [Bibr B31] [Bibr B32] [Bibr B33]). Also, no statistical difference for the serum level of MMP-1 protein was detected between RA cases and negative controls in the Chinese population (26 [Bibr B27] [Bibr B28] [Bibr B29]). Herein, we first enrolled a total of ten case-control studies for an integrative analysis, and found that high MMP-1 level in synovial fluid, but not serum, may be associated with the susceptibility to RA. In addition, two previous meta-analyses in 2015 reported the lack of genetic association between rs1799750 polymorphism of MMP-1 gene and risk of RA (38,39). This suggested the MMP-1 rs1799750 polymorphism maybe not contribute to the role of MMP-1 protein level in the risk of rheumatoid arthritis.

Some potential limitations should be noted in our study. A small sample size was included in some analyses. For instance, there were no more than ten case-control studies for the evaluation of the correlation between the MMP-1 level in the specific serum or synovial fluid and susceptibility to RA. A similar question exists in the stratification analyses of OA risk by the different sample sources, such as “cartilage tissue”, or “synovial fluid”. Thus, although we did not detect a correlation between the serum MMP-1 level and the risk of RA or OA, we still cannot rule out the possibility of association. In addition, the high heterogeneity in the Cohen statistics of RA or OA in the overall population, and a slight publication bias for the meta-analysis of RA could be a source of bias. Due to the sample collection feature of OA or RA, hospital-based controls were mainly included. More population-based controls are needed for the meta-analysis of MMP-1 serum level. Lastly, we only investigated the role of MMP-1 level in the risk of RA or OA. The combined effect of the expression levels of MMP-1 and other MMPs, such as MMP-2 and MMP-3, or the MMP expression and genetic variants, on the risk of RA or OA, should be investigated based on the available case-control studies in the future.

In summary, an elevated MMP-1 level in synovial fluid seemed to be related to a high risk of RA. Also, a high MMP-1 level in the cartilage tissue or synovial fluid may be associated with knee OA susceptibility in the Chinese population. A larger sample size is still required for the verification of our findings.

## Supplementary Material

Click here to view [pdf].
